# Imaging of Formaldehyde in Live Cells and *Daphnia magna* via Aza-Cope Reaction Utilizing Fluorescence Probe With Large Stokes Shifts

**DOI:** 10.3389/fchem.2018.00488

**Published:** 2018-10-15

**Authors:** Mingwang Yang, Jiangli Fan, Jianjun Du, Saran Long, Jia Wang, Xiaojun Peng

**Affiliations:** ^1^State Key Laboratory of Fine Chemicals, Dalian University of Technology, Dalian, China; ^2^Department of Breast Surgery, Institute of Breast Disease, Second Hospital of Dalian Medical University, Dalian, China

**Keywords:** fluorescence probe, formaldehyde, *Daphnia magna*, large Stokes shifts, Aza-Cope reaction, bioimaging

## Abstract

Formaldehyde (FA), a highly reactive carbonyl species, plays significant role in physiological and pathological functions. However, elevated FA will lead to cognitive impairments, memory loss and various neurodegenerative diseases due to its potent DNA and protein cross-linking mechanisms. In this work, a fluorescence probe, **BD-CHO**, based on benz-2-oxa-1, 3- diazole (**BD**) skeleton, was designed and synthesized for detection of FA via Aza-Cope reaction with high selectivity and large Stokes shifts (about 118 nm). **BD-CHO** was successfully applied to monitor the changes FA level in living cells, and kidney tissues of mice. Importantly it was the first time that **BD-CHO** was used for visualizing exogenous FA changes in *Daphnia magna* through fluorescence microscopy, demonstrating its potential application for studies of biological processes associated with FA.

## Introduction

Formaldehyde (FA) is a common environmental toxin but also endogenously produced through metabolism of amino acids or xenobiotics catalyzed by demethylases and oxidases, such as lysine-specific demethylase 1 (LDS1) (Shi et al., 2004) and semicarbazide-sensitive amine oxidase (SSAO) (O'Sullivan, 2004). The physiological FA levels ranging from 0.1 mM in blood to 0.4 mM intracellular (Andersen et al., 2010; Tong et al., 2013b) and is a well-established neurotoxin that affects memory, learning, and behavior (Tong et al., 2013a; Tulpule and Dringen, 2013). However, due to the rapidly growing list of modified DNA (Jones, 2012; Kohli and Zhang, 2013) and RNA (Jia et al., 2011) bases, elevated of FA are implicated in numerous disease pathologies, including neurodegenerative diseases, (Tong et al., 2011, 2013b) diabetes, heart disorders (Tulpule and Dringen, 2013), and Alzheimer's disease (Unzeta et al., 2007). Hence, the development of effective method for detecting FA in biosystem is urgent to understand the roles and metabolism process of FA.

Comparing to conventional detection approach, including colormetric assays (Luo et al., 2001), GC analyses (Bagheri et al., 2009; Chen et al., 2009), HPLC (Nash, 1953; Soman et al., 2008), fluorescence probes are considered as one of the most powerful tools due to their simplified operation, high selectivity and sensitivity, real-time detectability, and biocompatibility (Tang et al., 2015; Zhou et al., 2015; Bruemmer et al., 2017a; Xu et al., 2017). To date, a number of fluorescent probes for FA visualization have been reported based on the fluorophores of silicon rhodol, 1,8-naphthalimide, resorufin and so forth (Brewer and Chang, 2015; Roth et al., 2015; He et al., 2016; Bruemmer et al., 2017b; Liu et al., 2017; Xie et al., 2017a,b; Bi et al., 2018; Zhou et al., 2018). However, some of them suffered from disadvantages including time-consuming and rigorous synthetic procedure, high background fluorescence and small Stokes shifts which restricted their widely applications in biological system. In the presence of FA, partial probes encounter relatively small Stokes shifts which is not conducive to separating excitation and emission bands, in turn, cannot effectively minimize the interferences caused by self-absorption or auto-fluorescence (Abeywickrama et al., 2017; Chen et al., 2017). Meanwhile, low background fluorescence is beneficial for improving signal to noise ratio (SNR), facilitating to clearly visualize FA in complex biological environment. Hence, it is urgent to develop a FA-selective fluorescence probe with large Stokes shifts and low basal fluorescence.

**Figure 1:**
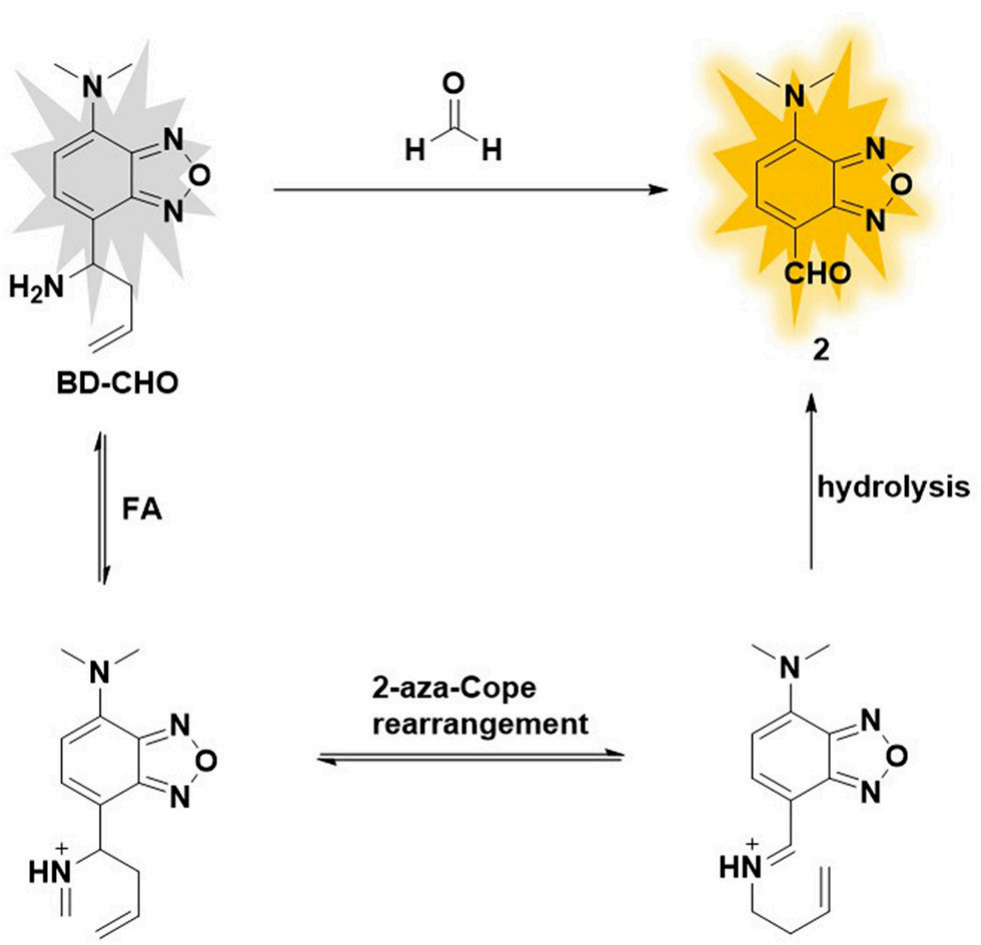
Design of formaldehyde probe BD-CHO.

Herein, we report an Aza-Cope reaction-based fluorescence probe (**BD-CHO**) for FA (Figure 1). The core structure of the probe was benz-2-oxa-1, 3- diazole (**BD**), an excellent fluorophore with facile modified and good stability which has been widely used to design fluorescence sensors (Taliani et al., 2007; Liu et al., 2011; Chen et al., 2012; Jiang et al., 2017), and homoallylic amine as FA-recognized group. **BD-CHO** shows good selectivity and sensitivity toward FA over other reactive carbonyl species (RCS) *in vitro* and is subsequently applied to visualize endogenous or exogenous FA in living cells and tissues. Significantly, to the best of our knowledge, **BD-CHO** is the first fluorescence probe for imaging FA in *Daphnia magna*.

## Experimental section

### Materials and instruments

Unless otherwise stated, all chemicals were purchased from commercial suppliers and used without further purification. The solvents were purified by conventional methods before used. All analytes were obtained by traditional methods. 4-Chloro-2, 1, 3-benzoxadiazole was purchased from TCI chemical. Silica gel (200–300 mesh) used for flash column chromatography was purchased from Qingdao Haiyang Chemical Co., Ltd. ^1^HNMR and ^13^CNMR spectra were determined by 400 MHz and 100 MHz using Bruker NMR spectrometers. Chemical shifts (δ) were expressed as parts per million (ppm, in CDCl_3_ or DMSO, with TMS as the internal standard). Meanwhile, high-resolution mass spectrometry was achieved with ESI-TOF and FTMS-ESI instrument. Fluorescence measurements were performed on an Agilent Technologies CARY Eclipse fluorescence spectrophotometer, and absorption spectra were measured on a PerkinElmer Lambda 35 UV–vis spectrophotometer. The pH values of sample solutions were measured with a precise pH-meter pHS-3C. Fluorescence quantum yield was achieved from a C11347-11 Absolute PL Quantum Yield Spectrometer. MTT assays were conducted on the Varioskan LUX Multimode Microplate Reader. The instrument used for imaging living cells and tissues of mice was an Olympus FV 1000 confocal microscopy purchased from Olympus.

### Determination of detection limits

According to the fluorescence titration data, a linear relationship between the fluorescence intensity (F _578__nm_) and FA concentrations was observed, the detection limit was calculated with the following equation: Detection limit = 3σ/k. Where σ is the standard deviation of blank measurements (*n* = 10), k is the slop between the fluorescence intensity vs. the concentrations of FA.

### Cytotoxicity assays

The MTT method was employed to assess the cellular cytotoxicity of **BD-CHO**. Before experiments, MCF-7 cells at a density of 1 × 10^4^ cells/well were seeded into 96-well plates and cultured for 24 h. Then the fresh culture contained **BD-CHO** over a range of concentrations (0–30 μM) (*n* = 6) to substitute the previous media, and further incubation for 24 h. After that, 10 μL of MTT (5 mg/mL in PBS) was added into per well and incubated another 4 h. Finally, 100 μL of DMSO was then added to dissolve formazan. The absorbance at 490 and 570 nm was measured in a microplate reader, and the cell viability (%) was calculated according to the following equation: Cells viability (%) = [OD570 (sample)—OD490 (sample)] / [OD570 (control)—OD490 (control)] × 100.

### Living cells incubation and imaging

MCF-7 cells, HepG2 cells and HeLa cells were purchased form Institute of Basic Medical Sciences (IBMS) of the Chinese Academy of Medical Sciences. MCF-7 cells were cultured in 90% Dulbecco's Modified Eagle Medium (DMEM, Gibico) supplemented with 10% FBS (Gibico) and 1% antibiotics (100 U/mL penicillin and 100 μg/mL streptomycin, Hyclone) in an atmosphere of 37°C and 5% CO_2_. One day before imaging, the cells were detached and were replanted on glass-bottomed dishes and allowed to adhere for 24 h. For imaging exogenous FA, the culture media of HepG2 cells was replaced with 2 mL of serum-free DMEM containing 10 μM fluorescent probe (from 2 mM stock in DMSO) and the cells were incubated for 30 min. Cells were then washed once with 2 mL PBS and then incubated further with FA (0.5 mM) for 3 h prior to imaging. For inhibition tests, FA-treated cells were incubated with DMEM containing sodium bisulfite (1 mM) and washed with PBS, then incubated with 10 μM fluorescent probe for 3 h before imaging. For imaging endogenous, MCF-7 cells were pretreated with or without (control) the inhibitor tranylcypromine (TCP) or GSK-LSD 1 for 20 h, followed by exchange into serum-free DMEM containing 10 μM fluorescent probe for 3 h.

### Fluorescence imaging of in kidney slices

Kidney slices were surgically exposed in Balb/c mice, which was approved by the Dalian Medical University Animal Care and Use Committee. The fresh kidney tissues were incubated with 10 μM **BD-CHO** for 30 min, and then, 1 mM FA was added for another 3 h. Before imaging, the tissues were washed with PBS three times. Olympus FV 1000 confocal microscopy with 20 × objective lens was used for fluorescence imaging. All of these experiments were carried out in accordance with the relevant laws and guidelines.

### Fluorescence imaging in *Daphnia magna*

The *D. magna* (age < 72 h) were cultured in clean non-chlorinated tap water under cool-white fluorescence light with light (14 h)-dark (10 h) photoperiod (Du et al., 2018). The animals were incubated with **BD-CHO** (10 μM) for 1 h, followed by washing twice with PBS and then incubated further with or without FA for 3 h. Olympus FV 1000 confocal microscopy with 4 × objective lens was used for fluorescence imaging.

### Synthesis of BD-CHO

**Figure 2:**
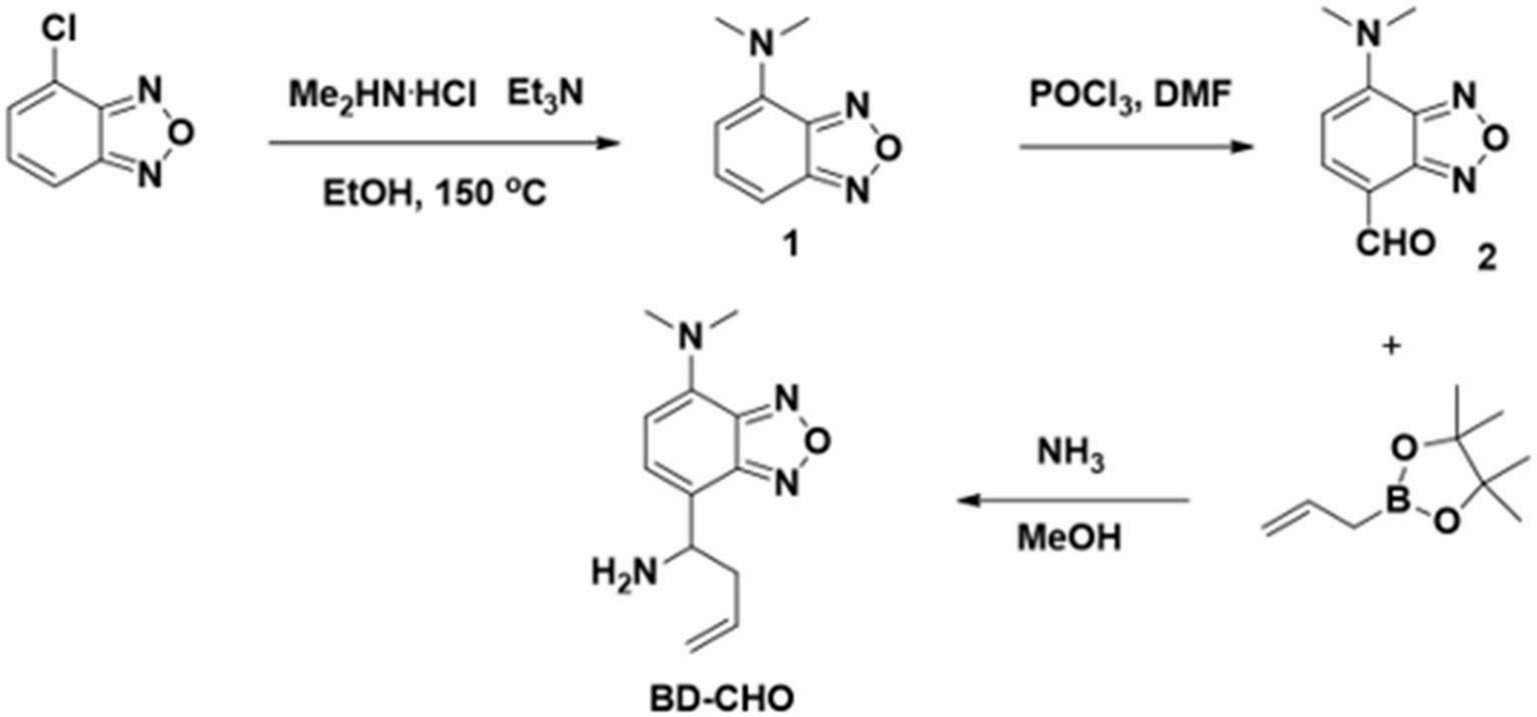
Synthetic procedure of BD-CHO.

The synthetic procedure was illuminated in Figure 2. Compound **1**: 4-Chloro-2,1,3-benzoxadiazole (1.0 g, 6.5 mmol), ethanol (10 mL), dimethylamine hydrochloride (3.0 g, 36.7 mmol), and triethylamine (6.0 mL) were mixed in a 25 mL autoclave at room temperature and followed by quick closure. Then, the bomb was heated with stirring at 150°C for 48 h. The mixture was cooled to room temperature and the solvent was removed under reduced pressure. After the addition of NaOH solution (2 M, 20 mL) to the residue, the mixture was extracted with ethyl acetate (30 Ml × 3). The combined organic layer was dried with anhydrous magnesium sulfate. After the removal of solvent, the product was purified by silica gel column chromatography with dichloromethane: petroleum ether (1:1) as the eluent to afford the desire product to yield red solid (965 mg, 91.0%). ^1^H NMR (400 MHz, CDCl_3_) δ (ppm): δ 7.18 (d, *J* = 7.3 Hz, 1H), 7.02 (s, 1H), 6.06 (s, 1H), 3.25 (s, 6H). ^13^C NMR (101 MHz, CDCl_3_) δ (ppm): 150.87, 145.72, 139.87, 133.36, 104.86, 102.13, 41.99, 1.01. MS (ESI-TOF): calculated for C_8_H_10_N_3_O^+^, [M+H]^+^, m/z, 164.08, found: 164.05.

Compound **2:** POCl_3_ (2 mL, 21 mmol) and anhydrous DMF (10 mL, 128 mmol) are mixed slowly with stirring in a round-bottomed flask at 0°C. Then, an anhydrous DMF (5 mL) containing compound **1** (960 mg, 5.9 mmol) was added into the mixture. Finally, the obtained mixture was stirred at room temperature about 6 h. The reaction was quenched by pouring the mixture into the ice water (50 mL). After pH was adjusted to pH ~ 9 by 10% NaOH, the mixture was extracted by ethyl acetate (30 mL × 3). The combined organic layer was dried with anhydrous magnesium sulfate. After the removal of solvent, the product was purified by silica gel column chromatography with petroleum ether: ethyl acetate (1:1) as the eluent to afford the desired product to yield red solid (743 mg, 65.8%). ^1^H NMR (400 MHz, CDCl_3_) δ (ppm): 10.03 (s, 1H), 7.89 (d, *J* = 8.2 Hz, 1H), 6.15 (d, *J* = 8.2 Hz, 1H), 3.58 (s, 6H). ^13^C NMR (101 MHz, CDCl_3_) δ (ppm): 185.93, 147.71, 145.08, 144.87, 142.15, 111.46, 102.35, 42.90, 1.01. HRMS (ESI-TOF): calculated for C_9_H_10_N_3_${\text{O}}_{2}^{+}$, [M+H]^+^, m/z, 192.0773, found: 192.0767.

### BD-CHO

To a solution containing compound **2** (192 mg, 1.0 mmol) in 20 mL of CH_3_OH, 6 mL of NH_3_ solution (7.0 M in CH_3_OH, 42 mmol) was added at 0°C under argon atmosphere and stirred 30 min. After that, allylboronic acid pinacol ester (0.48 mL, 2.5 mmol) was added, the mixture was warmed to ambient temperature and stirred overnight. The solvent was removed under reduced pressure, and the residue was purified by silica gel column chromatography with dichloromethane: methanol (50:1) as the eluent to afford probe **BD-CHO** as yellow solid (101 mg, 43.4%). ^1^H NMR (400 MHz, CDCl_3_) δ (ppm): 7.06 (d, *J* = 7.5 Hz, 1H), 5.94 (d, *J* = 7.6 Hz, 1H), 5.68 (dd, *J* = 17.0, 9.7 Hz, 1H), 4.99 (dd, *J* = 19.8, 13.8 Hz, 2H), 4.20 (t, *J* = 6.6 Hz, 1H), 3.19 (s, 6H), 2.71–2.54 (m, 1H), 2.48 (dd, *J* = 14.2, 7.2 Hz, 1H), 2.09 (s, 2H). ^13^C NMR (101 MHz, CDCl_3_) δ (ppm) 149.25, 146.08, 138.78, 135.27, 129.21, 120.35, 117.72, 105.15, 52.63, 41.94, 41.81. HRMS (ESI-TOF): calculated for C_12_H_12_N_3_O^−^, [M-NH_2_]^−^, m/z, 216.1137, found: 216.1134.

## Results and discussion

### Design and synthesis of BD-CHO

To design selective and sensitive fluorescence probe for the FA detection, we focus on homoallylamine trigger which can specially react with FA and produce an electron-withdrawing aldehyde group via an Aza-Cope rearrangement reaction. Benz-2-oxa-1, 3- diazole (**BD**) was chosen as fluorophore core due to easily regulated intramolecular charge transfer (ICT) and excellent photophysical property. In this probe, dimethylamine group was introduced as an electron-donating group (EDG) and homoallylic amine as FA-recognized group. The original **BD-CHO** exhibits almost no fluorescence due to the poor electron withdrawing ability of the homoallylamine moiety. After addition of FA, the amine reacts selectively with FA to form an imine intermediate, which simultaneously undergoes 2-Aza-Cope rearrangement and final hydrolysis to produce an aldehyde group with power electron withdrawing ability (Figure 1) (Brewer et al., 2017; Dou et al., 2017). As a result, the intramolecular charge transfer (ICT) process from π-conjugated electron donor (dimethylamine group) to the aldehyde group is opened, showing a “turn-on” fluorescence response.

The probe **BD-CHO** was readily prepared by coupling compound **2** with allylboronic acid pinacol ester in the presence of NH_3_ solution. The intermediates and target compound were well characterized by ^1^H NMR, ^13^C NMR, HR-MS (Figures S11–S16).

### Spectral properties of BD-CHO

**Figure 3:**
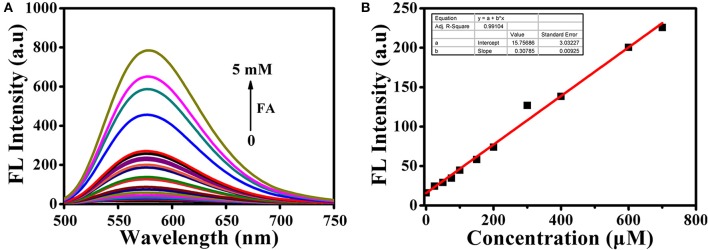
**(A)** Fluorescence spectra of 10 μM BD-CHO after addition of FA in HEPES buffer (20 mM, pH 7.4, 50% DMSO). **(B)** Linear relationship between the fluorescence intensity of BD-CHO (10 μM) and FA concentration (0–800 μM) in HEPES buffer (20 mM, pH 7.4, 50% DMSO). λ_ex_ = 460 nm, slit: 10/10 nm.

All the spectra of **BD-CHO** were investigated in HEPES buffer containing DMSO (V/V = 1:1, 20 mM, pH 7.4). Upon addition of FA, the maximum absorption wavelength shifted from 450 to 460 nm (Figure S1). The probe exhibited almost no fluorescence in the absence of FA, which made **BD-CHO** more favorable probe for highly sensitive detecting FA. In the presence of 5 mM FA, however, the fluorescence intensity sharp enhanced about 55-fold at 578 nm with excited at 460 nm (Figure 3A) and the fluorescence quantum yield increased from 0.015 to 0.080. Importantly, **BD-CHO** displayed a large Stokes shifts (118 nm) in response to FA (Figure S2), which is beneficial for fluorescent detection in view of reduction of self-absorption. Upon addition of 5 mM FA, the fluorescence intensity of **BD-CHO** increased dramatically over 50 min and reaches a plateau after approximately 3 h (Figure S3). There was a linear concentration-dependent fluorescent response with **BD-CHO** toward FA ranging from 0 to 0.8 mM, with a high correlation coefficient *R*^2^ = 0.991 (Figure 3B). Thus, the detection limit was calculated to be around 9.7 μM, allowing its applicability for the detection of the intracellular FA (100–400 μM) (Andersen et al., 2010).

### Selectivity and pH influence

**Figure 4:**
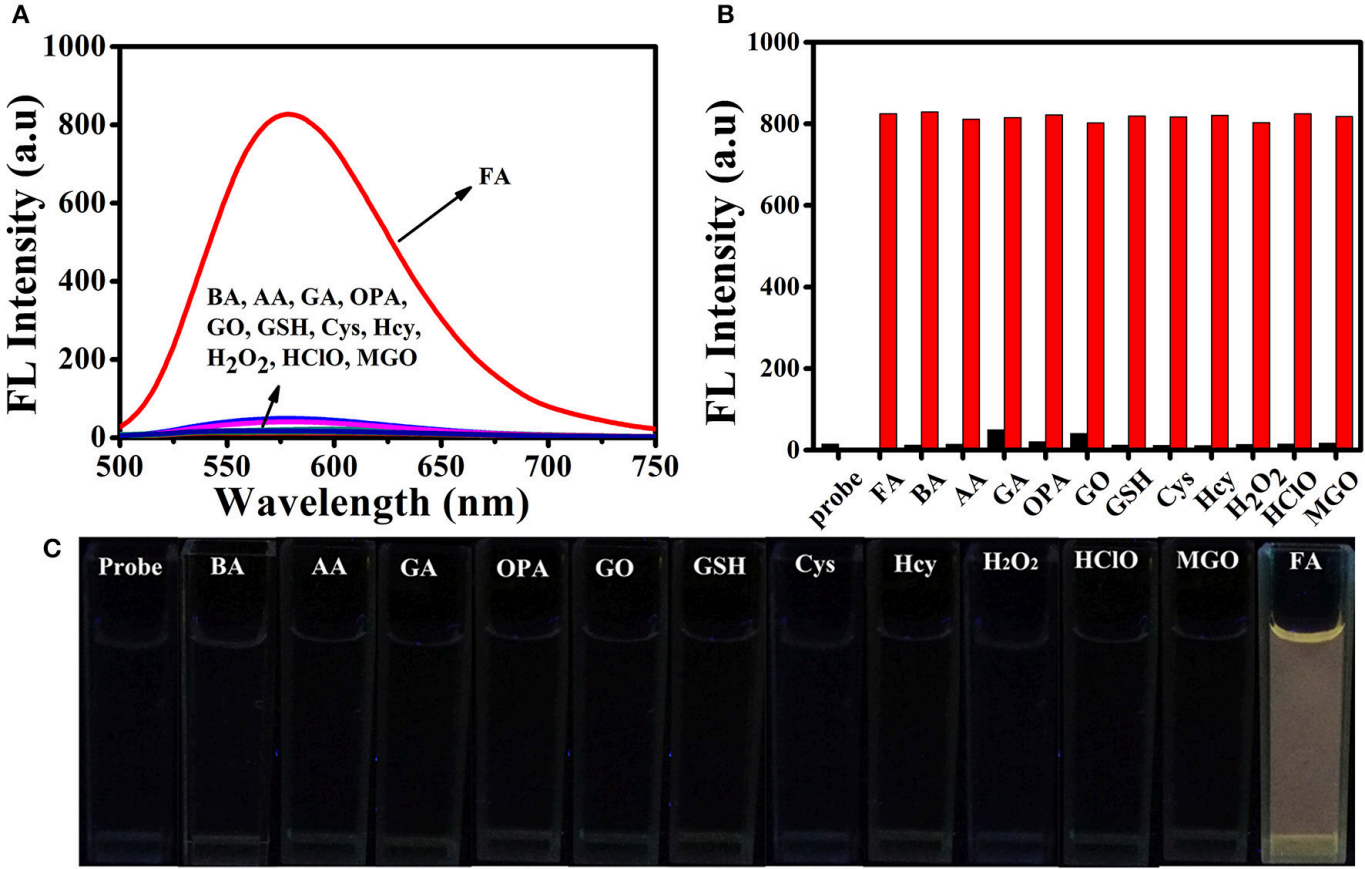
**(A)** Fluorescence spectra of 10 μM BD-CHO in the presence of FA (5 mM) and other various analytes (5 mM) in HEPES buffer (20 mM, pH 7.4, 50% DMSO). **(B)** Fluorescence intensity of BD-CHO (at 578 nm) in the presence of various analytes (black) or in the presence of both FA and the corresponding analyte (red): Formaldehyde (FA), benzaldehyde (BA), acetaldehyde (AA), glutaraldehyde (GA), *o*-phthalaldehyde (OPA), glyoxal (GO), Glutathione (GSH), Cysteine (Cys), Homo cysteine (Hcy), H_2_O_2_, HClO, Methylglyoxal (MGO) in HEPES buffer (20 mM, pH 7.4, 50% DMSO). λ_ex_ = 460 nm, slit: 10/10 nm. **(C)** The color fluorescence images of BD-CHO in FA and other analytes under illumination with a 365 nm UV lamp.

**Figure 5:**
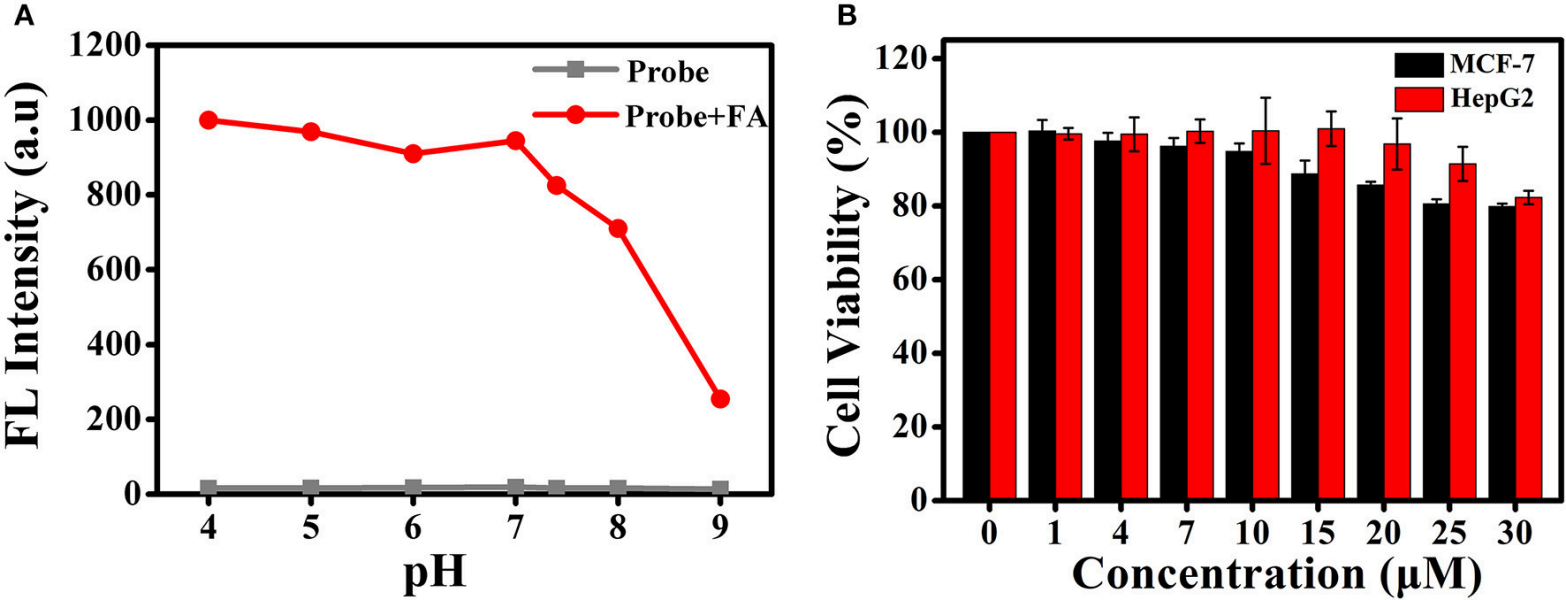
**(A)** Fluorescence responses of BD-CHO (10 μM) in the presence (red) and absence (blank) of FA in HEPES buffer (20 mM, pH 7.4, 50% DMSO) with different pH values. λ_ex_ = 460 nm, slit: 10/10 nm **(B)** The cell cytotoxicity of BD-CHO in MCF-7 and HepG2 cells.

High selectivity is necessary and crucial for evaluating the applicability of the fluorescent probe. Therefore, we investigated the ability of **BD-CHO** to distinguish FA from various relevant species, including benzaldehyde (BA), acetaldehyde (AA), glutaraldehyde (GA), *o*-phthalaldehyde (OPA), glyoxal (GO), methylglyoxal (MGO), biothiols (Cys, Hcy, and GSH), H_2_O_2_, HClO. As shown in Figure 4A, negligible fluorescence intensity changes of **BD-CHO** were obtained in the presence of possible interfering analytes but a 55-fold fluorescence enhancement with FA. The fluorescence change can also be observed with naked eyes under illumination with a 365 nm UV lamp (Figure 4C). To further evaluate the selectivity, competitive experiments were performed in the presence of 5 mM FA and various other species (Figure 4B). **BD-CHO** still responded to FA with turn-on fluorescence signal in the presence of competitive species. These results demonstrate that the ability of **BD-CHO** to specifically recognize FA over others relative analytes in complexed biological system. Subsequently, we investigated the effect of pH on the recognition of FA. As shown in Figure 5A, fluorescence response of **BD-CHO** to FA is independent of pH in the range 5.0–8.0, indicating its suitability for imaging under physiological conditions.

### Recognized mechanism

**Figure 6:**
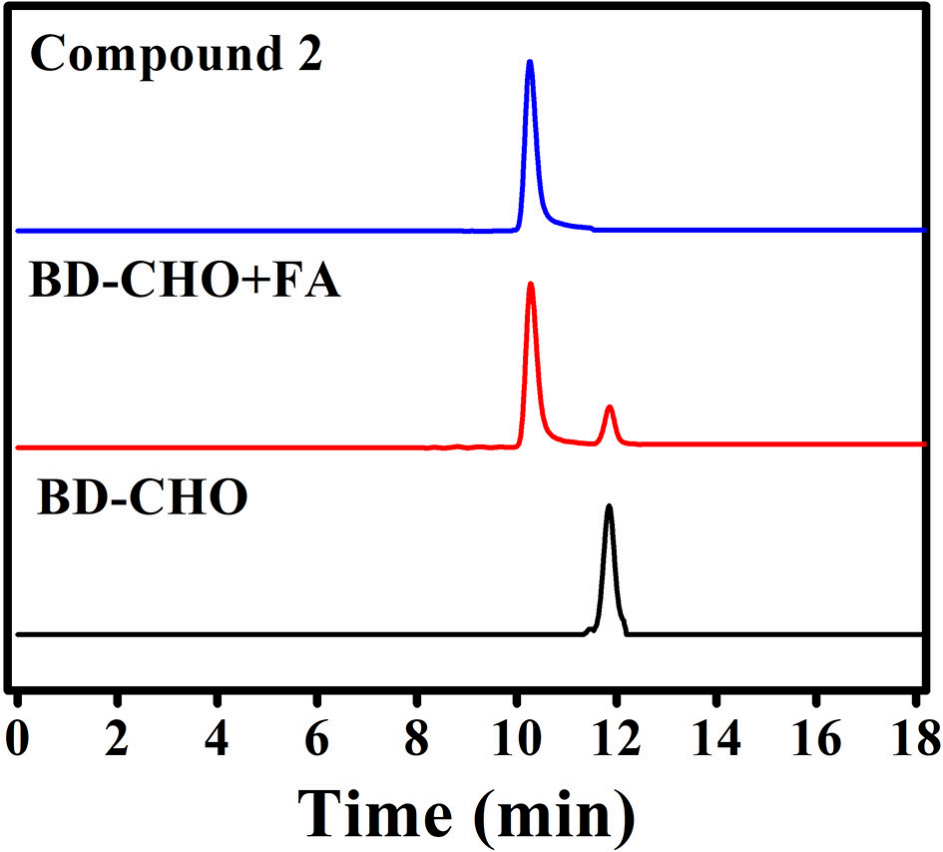
HPLC analysis of compound 2, probe BD-CHO before (black) and after (red) incubation with FA (5 mM) at 37°C for 2 h.

The possible sensing mechanism was shown in Figure 1. Firstly, homoallylamine moiety in **BD-CHO** reacted with FA to afford 2-aza-1,5-dienes, and then the 2-Aza-Cope rearrangement occurred via [3,3]-migration to form α, β-ene, which was hydrolyzed in aqueous solution to release highly fluorescent compound **2**. In order to verify the proposed hypothesis, we compared the absorption and fluorescence spectra of **BD-CHO** in the presence of FA with that of the compound 2. As shown in Figure S4, the absorption and fluorescence spectra of **BD-CHO** + FA were almost identical to that of compound **2**, indicating that compound **2** might be the final reaction product of the **BD-CHO** with FA. Meanwhile, HPLC analysis was used to further confirm the detected product. As shown in Figure 6, the chromatographic peak of compound **2** and **BD-CHO** were found at 10.27 and 11.85 min, respectively. Additionally, upon addition of FA (5 mM) and incubation with **BD-CHO** for 2 h, the reaction product exhibited a chromatographic peak at 10.27 min, which matched perfectly with compound 2; a weak peak was also observed at 11.85 min, corresponding to probe **BD-CHO**. Furthermore, HR-MS confirmed this result, where a noticeable signal peak at m/z = 214.0584 was assigned to [M + Na]^+^ (compound **2**, calculated for 214.0592) (Figure S5). This is consistent with the previously reported mechanism (Xu et al., 2016; Yang et al., 2018).

### Fluorescence imaging of exogenous and endogenous FA in living cells

Encouraged by the excellent photophysical properties of **BD-CHO** and its selective response to FA *in vitro*, we attempted to assess the suitability of **BD-CHO** for monitoring FA in living cells. Accordingly, the cytotoxicity of **BD-CHO** was established using MTT assays with MCF-7 cells and HepG 2 cells. It was found that the cell viabilities exceed 94% when incubated cells for 24 h with 10 μM **BD-CHO**, demonstrating the low cytotoxicity of **BD-CHO** (Figure 5B). The probe has good stability in biological medium (Figure S6).

**Figure 7:**
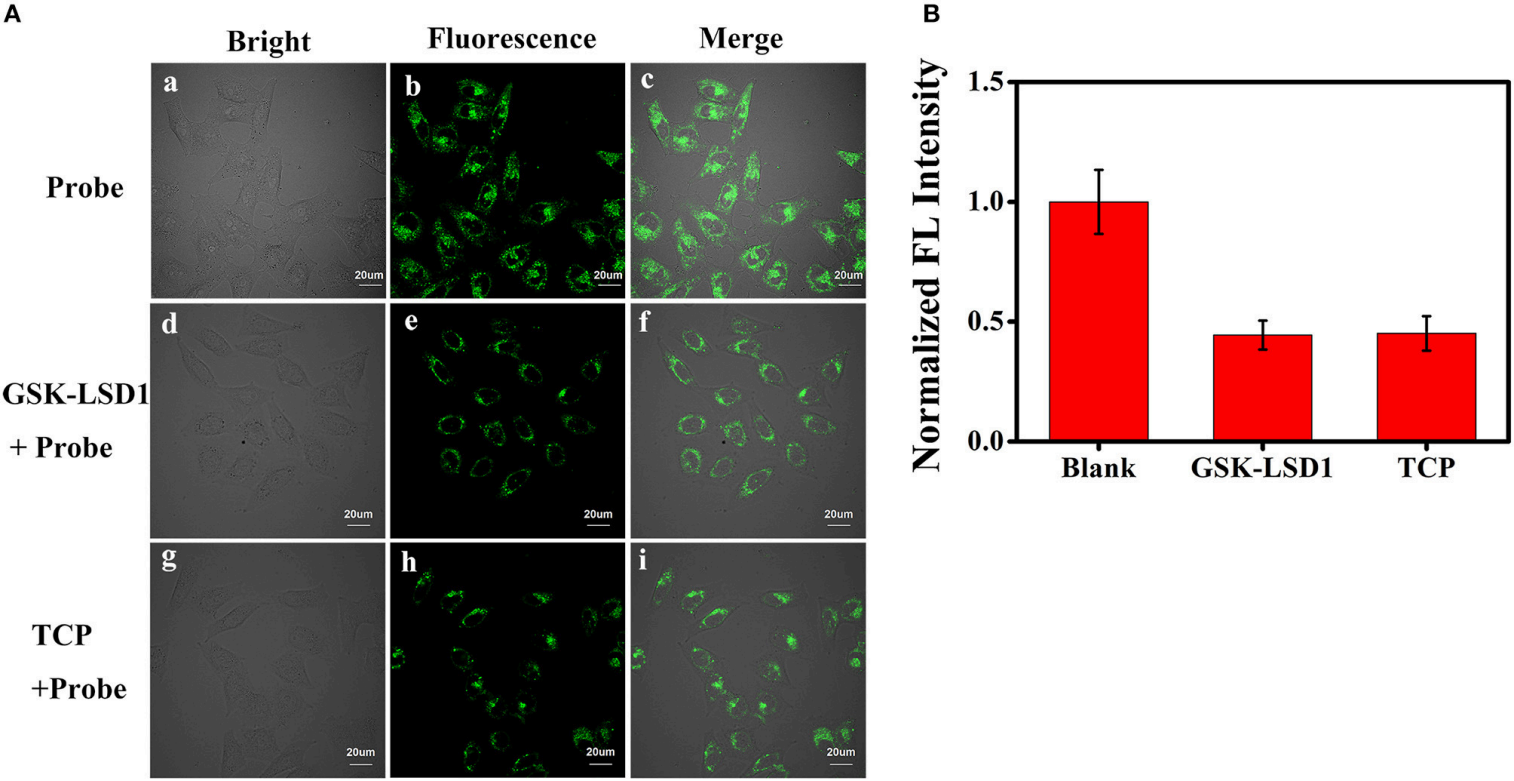
**(A)** Fluorescence image for BD-CHO in GSK-LSD1 and TCP treated MCF-7 cells. (a-c) blank, (d-f) Cells were pre-treated with 1 μM GSK-LSD1 for 20 h and then with BD-CHO (10 μM) for 3 h, (g-i) Cells were pre-treated with 20 μM TCP for 20 h and then with BD-CHO (10 μM) for 3 h. λ_ex_ = 488 nm, collected 550–600 nm. **(B)** Mean fluorescence intensity of MCF-7 cells treated with blank, GSK-LSD1 and TCP. Scale bar: 20 μm.

Subsequently, we evaluated the ability of **BD-CHO** to visualize changes of FA in living cells using confocal microscopy. HepG2 cells were treated with 10 μM **BD-CHO** for 30 min at 37°C, it showed almost invisible fluorescence signal when excited at 488 nm (Figures S7a–c). When probe loaded cells were treated with 0.5 mM FA for another 3 h, obvious green fluorescence signal was observed (Figures S7d–f). NaHSO_3_ was used for negative experiments, because it can efficiently react with FA to destroy the central carbonyl group (Tang et al., 2016). When the cells were pre-treated with 0.5 mM FA and 1 mM NaHSO_3_ for 30 min, and then cultured with **BD-CHO** for 3 h, the green fluorescence became faint (Figures S7g–i). Owing to overexpression of LSD1, MCF-7 cells were known to showed elevated FA levels (Liu et al., 2013). When the LSD1 was pharmacological inhibited, the FA levels significantly decreased (Brewer and Chang, 2015). The MCF-7 cells were treated with **BD-CHO** and the cells exhibited strong green fluorescence signal (Figure 7Ab), which indicated a high level of FA in MCF-7 cells. However, when MCF-7 cells were incubated with 1 μM GSK-LSD1 (Munoz, 2015) (an LSD1 inhibitor with an IC_50_ of 42 nM) and then with **BD-CHO**, a decrease in **BD-CHO** fluorescence signal compared to control cells was observed (Figure 7Ae). Additionally, treatment with 20 μM tranylcypromine (Lee et al., 2006) (TCP, an LSD1 inhibitor with an IC_50_ of 2 μM), also attenuated **BD-CHO** fluorescence (Figures 7Ah,B). Meantime, the detection of endogenous FA changes in HeLa cells were also carried out (Figure S8). Take together, the data showed that **BD-CHO** is capable of detecting exogenous and endogenous produced FA in living cells.

### Fluorescence imaging in kidney tissues

**Figure 8:**
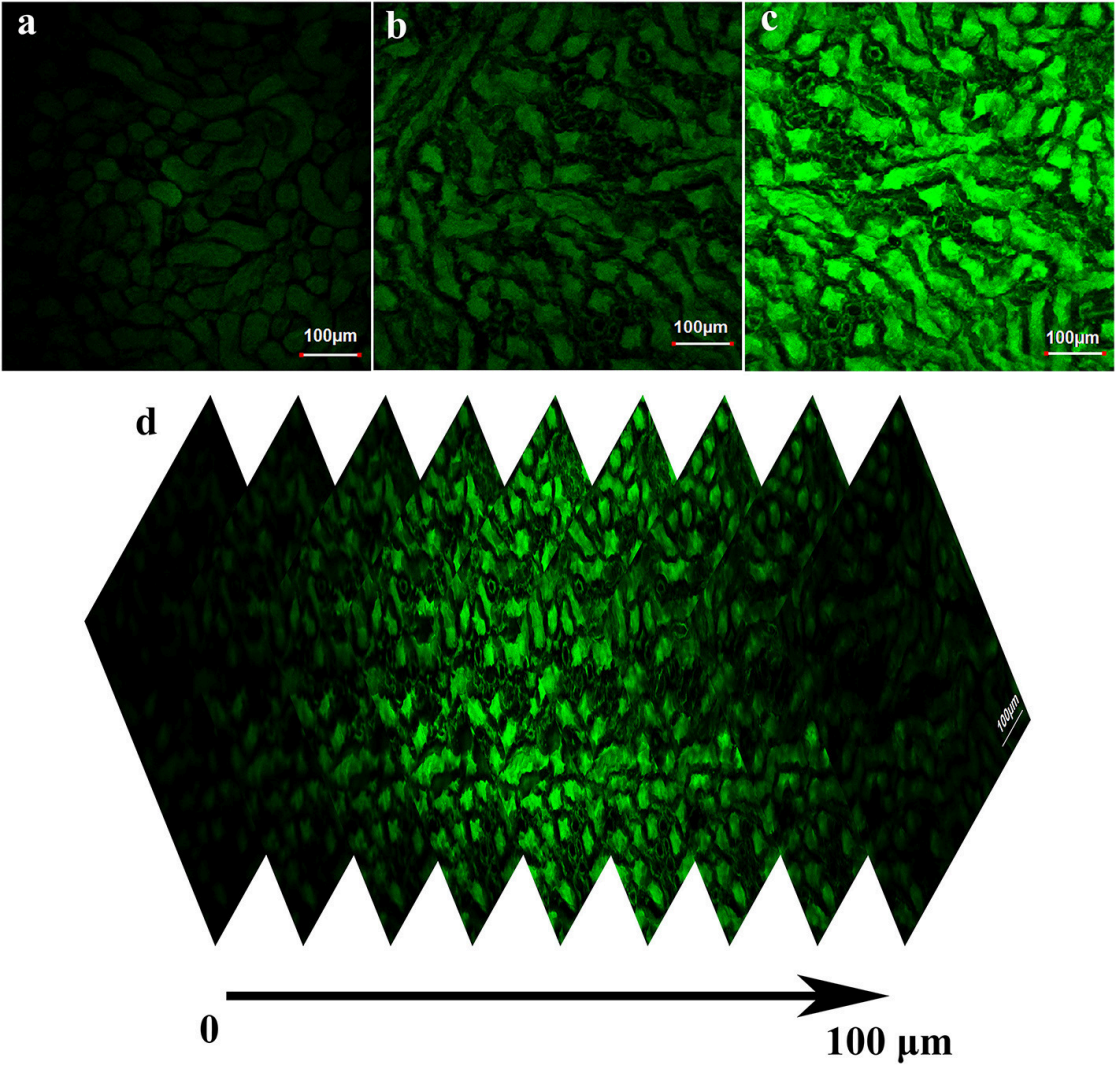
Fluorescence image of fresh kidney tissue slices stained with 10 μM BD-CHO for 1 h and then treated without **(a)** and with **(b)** 0.5 mM **(c)** 1 mM FA for further 3 h. **(d)** Fluorescence image of kidney tissue slices incubated with BD-CHO and FA at different depths (0–100 μm). λ_ex_ = 488 nm, collected 550–600 nm. Scale bar: 100 μm.

We further investigated whether **BD-CHO** could image FA in living kidney tissue slices. The kidney tissue slices only soaked in 10 μM **BD-CHO** solution for 3 h showed negligible fluorescence signal (Figure 8a, Figure S9). By contrast, when the mice kidney tissue slices were soaked in 10 μM **BD-CHO** solution for 30 min, and then soaked in the FA solution for another 3 h, the strong green fluorescence signals were observed (Figures 8b,c) with the penetration depth of up to about 100 μm (Figure 8d, Figure S10). These results indicated that **BD-CHO** was capable of imaging FA in the kidney tissue slices.

### Fluorescence imaging in *Daphnia magna*

**Figure 9:**
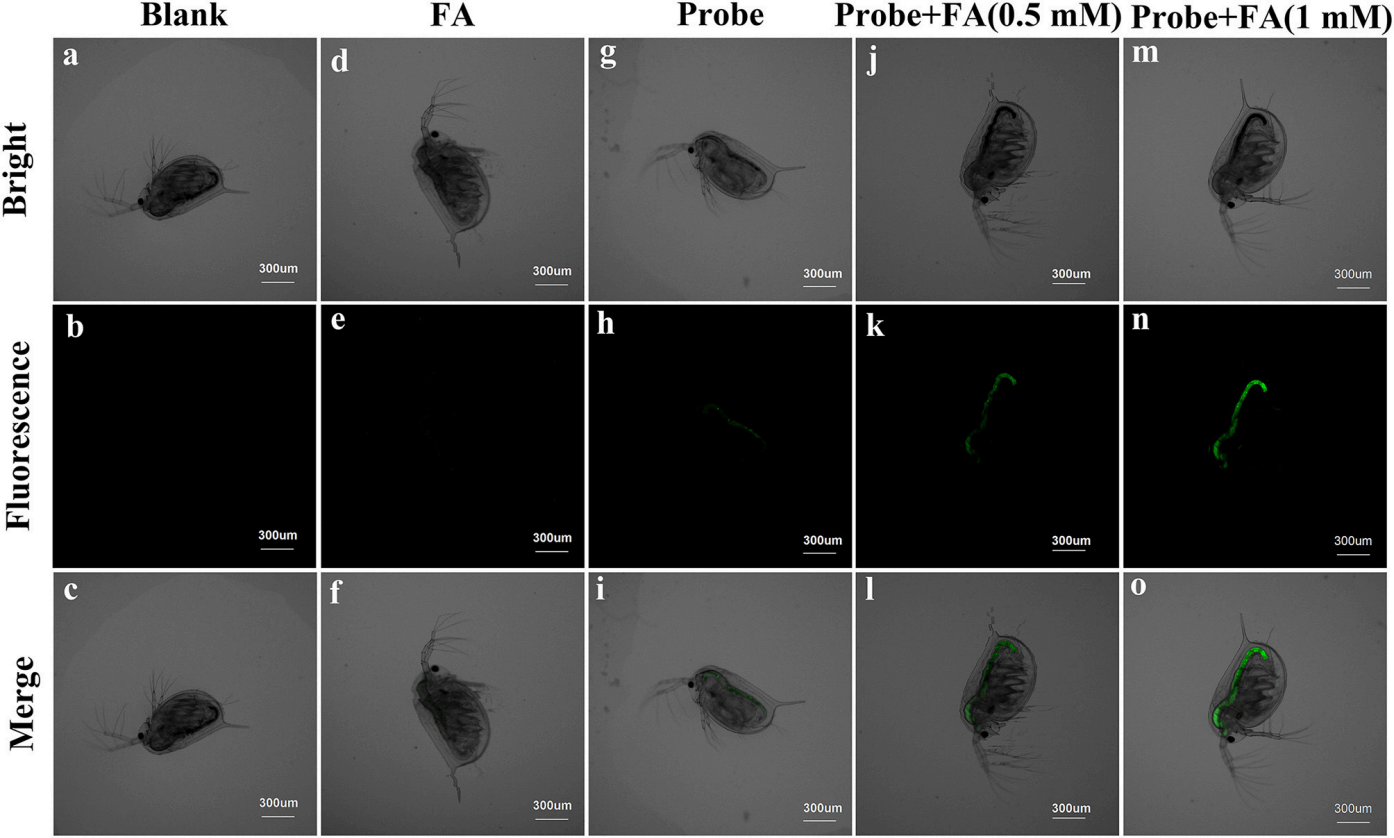
Fluorescence imaging of living *Daphnia magna. Daphnia magna* treated with PBS **(a–c)**, FA **(d–f)** and probe **(g–i)** independently. *Daphnia magna* were incubated with 10 μM BD-CHO for 1 h and then with 0.5 mM **(j–l)** and 1 mM **(m–o)** FA for another 3 h before imaging. λ_ex_ = 488 nm, collected 550–600 nm. Scale bar: 300 μm.

The ability of **BD-CHO** for detecting FA *in vivo* was evaluated in living *Daphnia magna*, a widely used animal as a standard Environmental Protection Agency test organism (Lovern and Klaper, 2006), using fluorescence imaging. The untreated and FA-treated *Daphnia magna* showed no fluorescence signal (Figures 9a–f). It exhibits faint green fluorescence signal when the *Daphnia magna* were incubated with 10 μM **BD-CHO** for 3 h at 25°C (Figures 9g–i). By contrast, upon in the succession treated with **BD-CHO** and different concentration FA, the *Daphnia magna* displays noticeable fluorescence enhancement in green channel (Figures 9j–o), indicating that **BD-CHO** could be used for the fluorescence imaging of FA in living *Daphnia magna*.

## Conclusions

In summary, an efficient fluorescent probe, **BD-CHO**, for selective detection of FA via 2-Aza-Cope reaction with large Stokes shifts has been designed and synthesized. In the presence of FA, the fluorescence intensity was significantly increased (about 55-fold) and exhibited large Stokes shifts (about 118 nm). The recognition mechanism of **BD-CHO** to FA was confirmed by HPLC and MS analysis. The probe was used for the fluorescence imaging of exogenous and endogenous FA in living cells with low cytotoxicity and autofluorescence. In addition, **BD-CHO** could also detect FA in living kidney tissue slices with a penetration depth of up to about 100 μm. More importantly, **BD-CHO** was successfully applied to monitor exogenous FA changes in *Daphnia magna* for the first time. All the results indicated that **BD-CHO** could potentially serve as a useful tool for studying the pathology and physiology role of FA in complex biosystem.

## Ethics statement

This study was carried out in accordance with the recommendations of Dalian Medical University Animal Care and Use Committee. The protocol was approved by the Dalian Medical University Animal Care and Use Committee.

## Author contributions

MY was responsible for designing and performing the experiments. JD and SL were responsible for the characterization of compounds. JF, JW, and XP were responsible for discussing and revising the paper.

### Conflict of interest statement

The authors declare that the research was conducted in the absence of any commercial or financial relationships that could be construed as a potential conflict of interest.
