# Supplementary material for: Imaging of Formaldehyde in Live Cells and Daphnia magna via Aza-Cope Reaction Utilizing Fluorescence Probe With Large Stokes Shifts
Source: Front Chem. 2018 Oct 15;6:488. doi: 10.3389/fchem.2018.00488 (PMC6196232; doi:10.3389/fchem.2018.00488)
Supplement: Supplementary file 1 [file Table_1.DOCX]

Supplementary Material

Imaging of formaldehyde in live cells and *Daphnia magna* via Aza-Cope reaction utilizing fluorescence probe with large Stokes shifts

Mingwang Yang^1^, Jiangli Fan^1*^, Jianjun Du^1^, Saran Long^1^, Jia Wang^2*^, Xiaojun Peng^1^

*** Correspondence:** Jiangli Fan: fanjl@dlut.edu.cn

Jia Wang: wangjia0829jp@yahoo.co.jp





**Figure S1** Absorption spectra of **BD-CHO** in HEPES buffer (20 mM, pH 7.4, 50% DMSO) with (red) or without (black) FA.


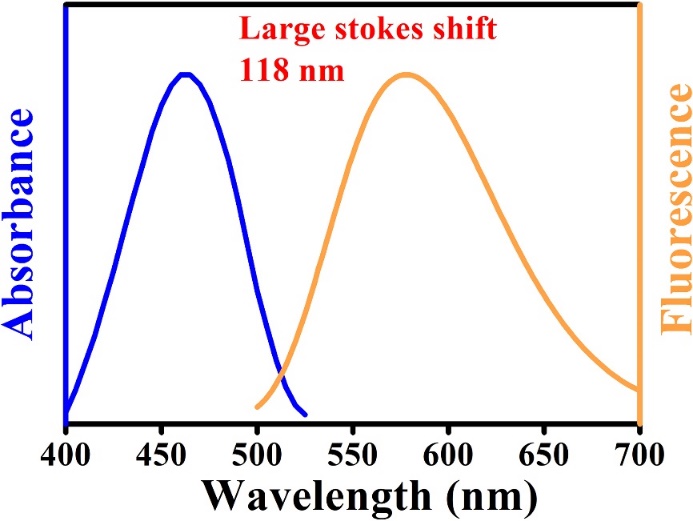


**Figure S2** Normalized absorption and fluorescence spectra of 10 μM **BD-CHO** in presence of FA in HEPES buffer (20 mM, pH 7.4, 50% DMSO).


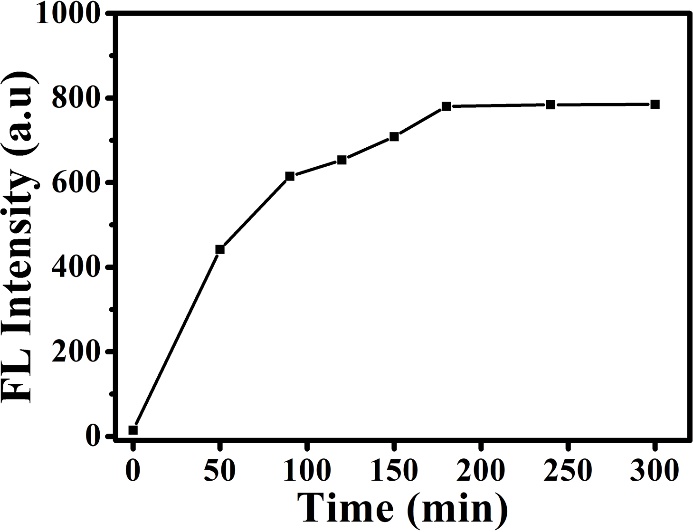


**Figure S3** Time dependent fluorescence intensity changes against reaction time of BD-CHO toward FA


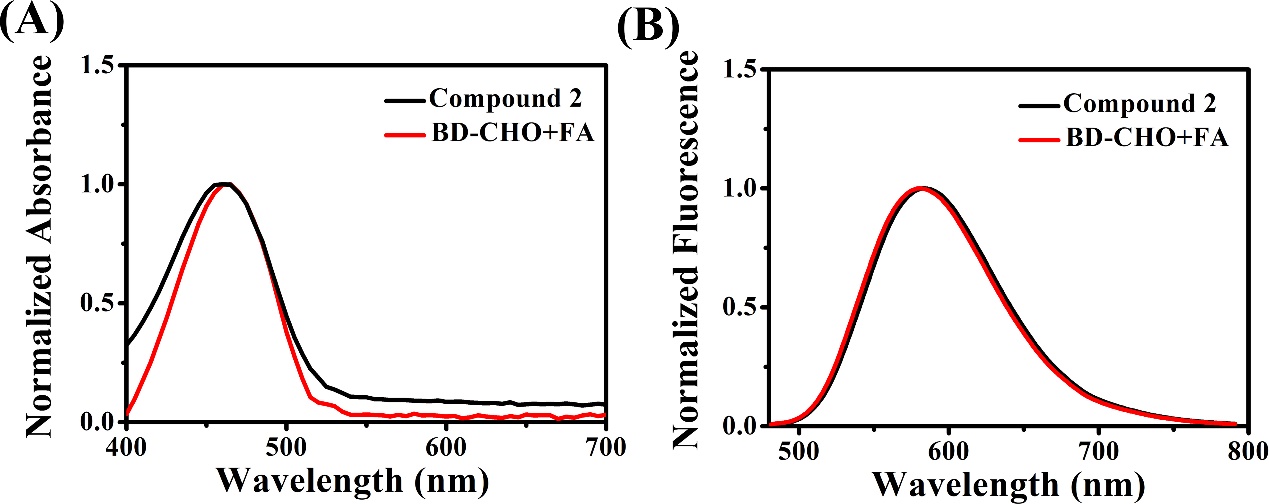


**Figure S4** Normalized absorbance and fluorescence spectra of 10 μM Compound **2** and **BD-CHO** in presence of FA under the HEPES buffer (20 mM, pH 7.4, 50% DMSO). λ_ex_=460 nm, slit: 10/10 nm


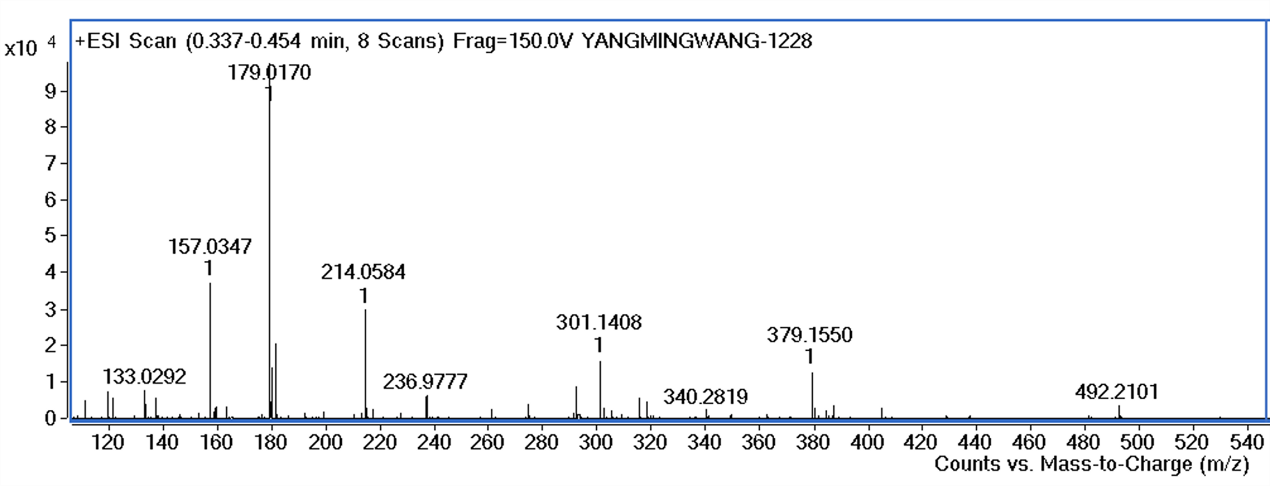


**Figure S5** HRMS of **BD-CHO** after addition of FA.


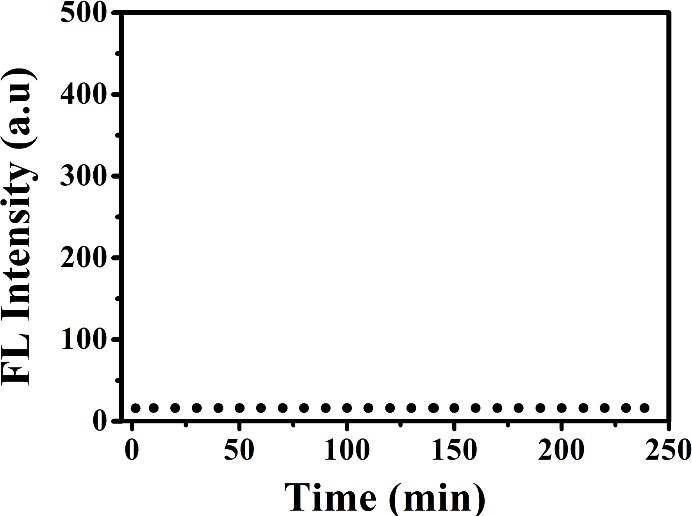


**Figure S6** The stability of the BD-CHO in DMEM.


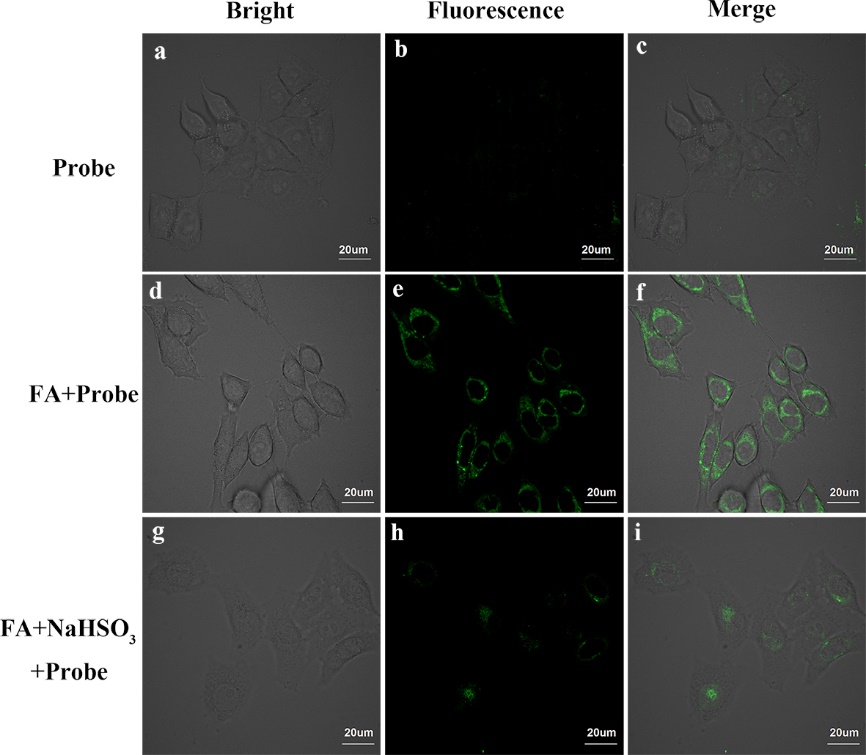


**Figure S7** Fluorescence imaging for **BD-CHO** in HepG2 cells. Cells were treated with (a-c) 10 μM **BD-CHO** for 30 min, then incubated with DMEM for another 3 h; (d-f) 10 μM **BD-CHO** for 30 min, then with 0.5 mM FA for another 3 h; (g-i) 0.5 mM FA and 1 mM NaHSO_3_ for 30 min, and then cultured with **BD-CHO** for 3 h. λ_ex_=488 nm, collected 550-600 nm. Scale bar: 20 μm.


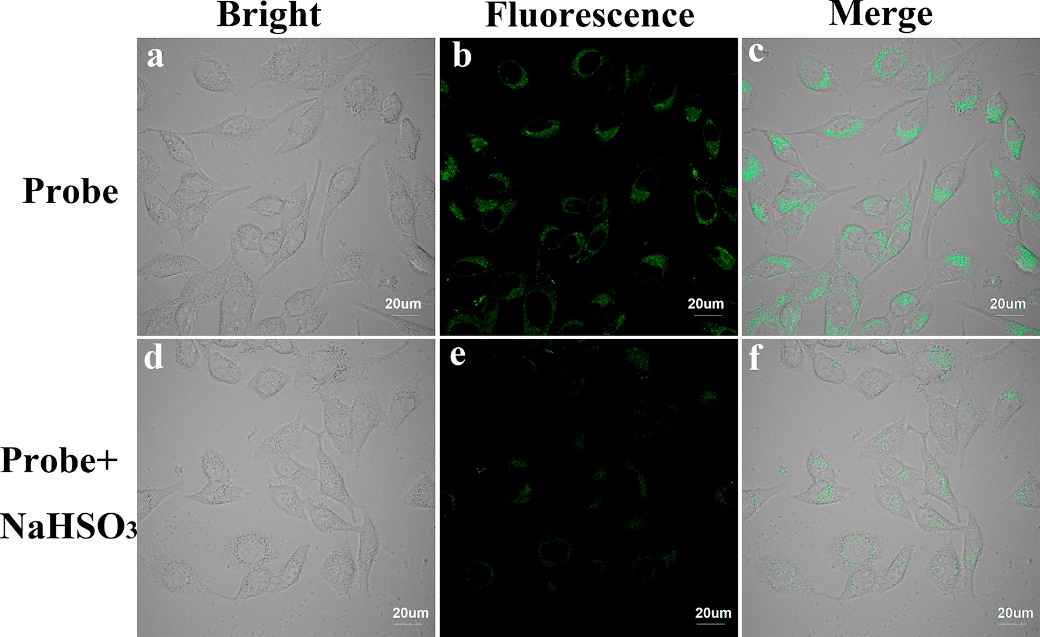


**Figure S8** Fluorescence imaging for **BD-CHO** in HeLa cells. Cells were treated with (a-c) 10 μM **BD-CHO** for 30 min, then incubated with DMEM for another 3 h; (d-f) 1 mM NaHSO_3_ for 30 min, and then cultured with **BD-CHO** for 3 h. λ_ex_=488 nm, collected 550-600 nm. Scale bar: 20 μm.


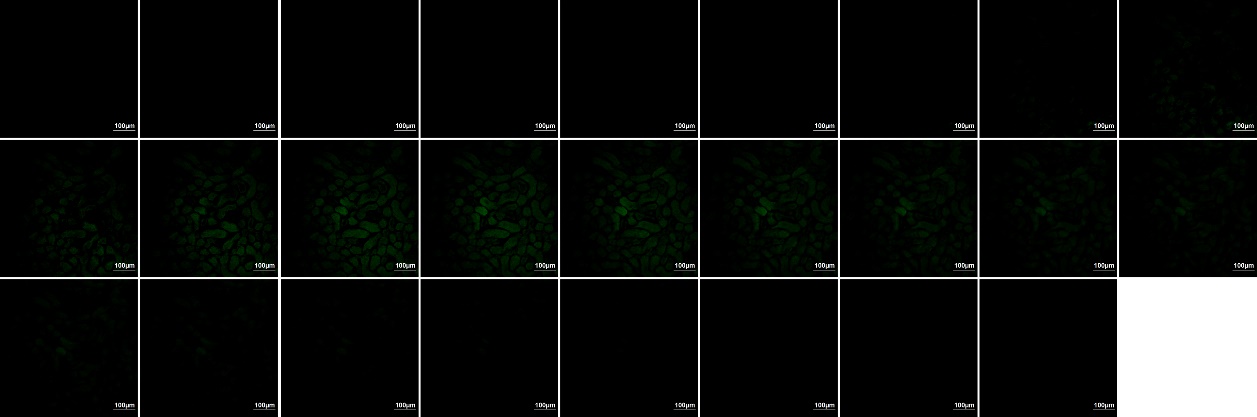


**Figure S9** Depth fluorescence image of a mice kidney tissue slice (0-100 μm). The slice was pretreated with 10 μM **BD-CHO** for 30 min and washed with PBS, the further incubated with 3 h. Step size: 4 μm. λ_ex_=488 nm, collected 550-600 nm. Scale bar: 100 μm.


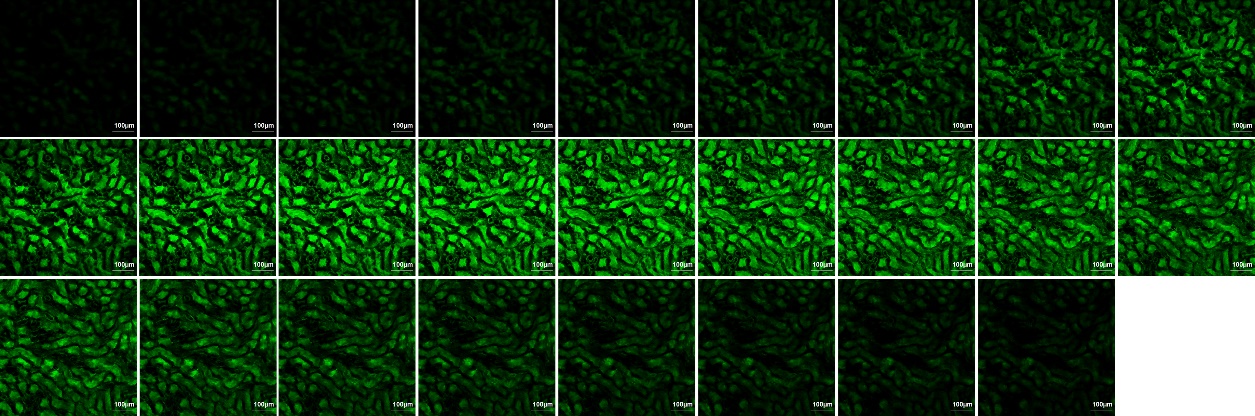


**Figure S10** Depth fluorescence image of a mice kidney tissue slice (0-100 μm). The slice treated with 10 μM **BD-CHO** for 30 min, then with 1 mM FA for another 3 h. Step size: 4 μm. λ_ex_=488 nm, collected 550-600 nm. Scale bar: 100 μm.


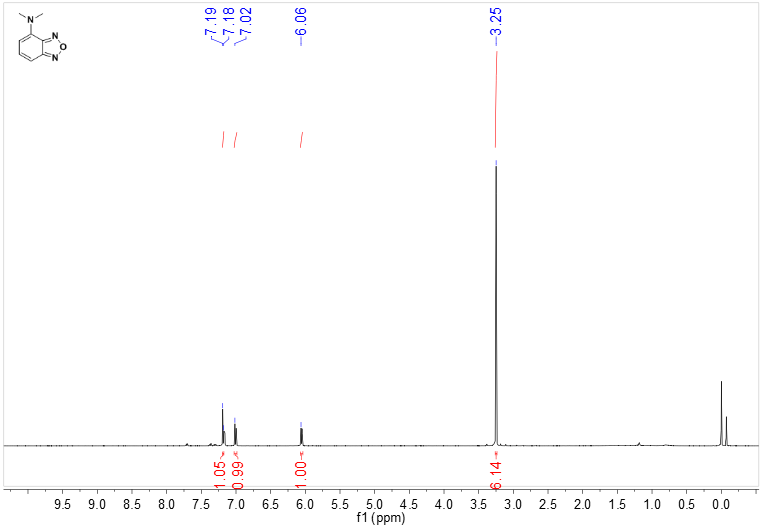


**Figure S11** ^1^H NMR of the compound **1** in CDCl_3_


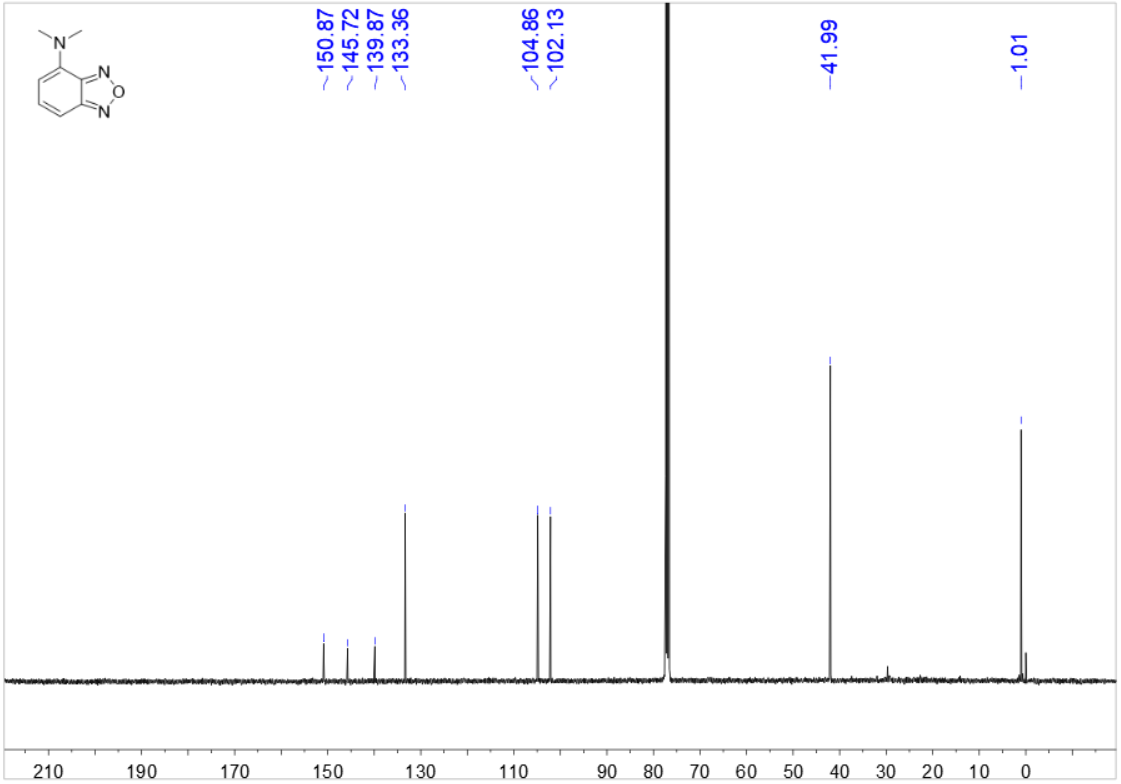


**Figure S12** ^13^C NMR of the compound **1** in CDCl_3_


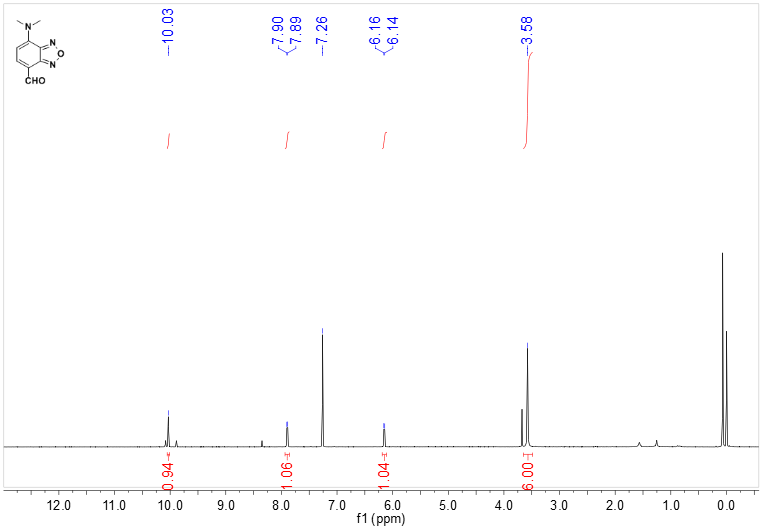


**Figure S13** ^1^H NMR of the compound **2** in CDCl_3_


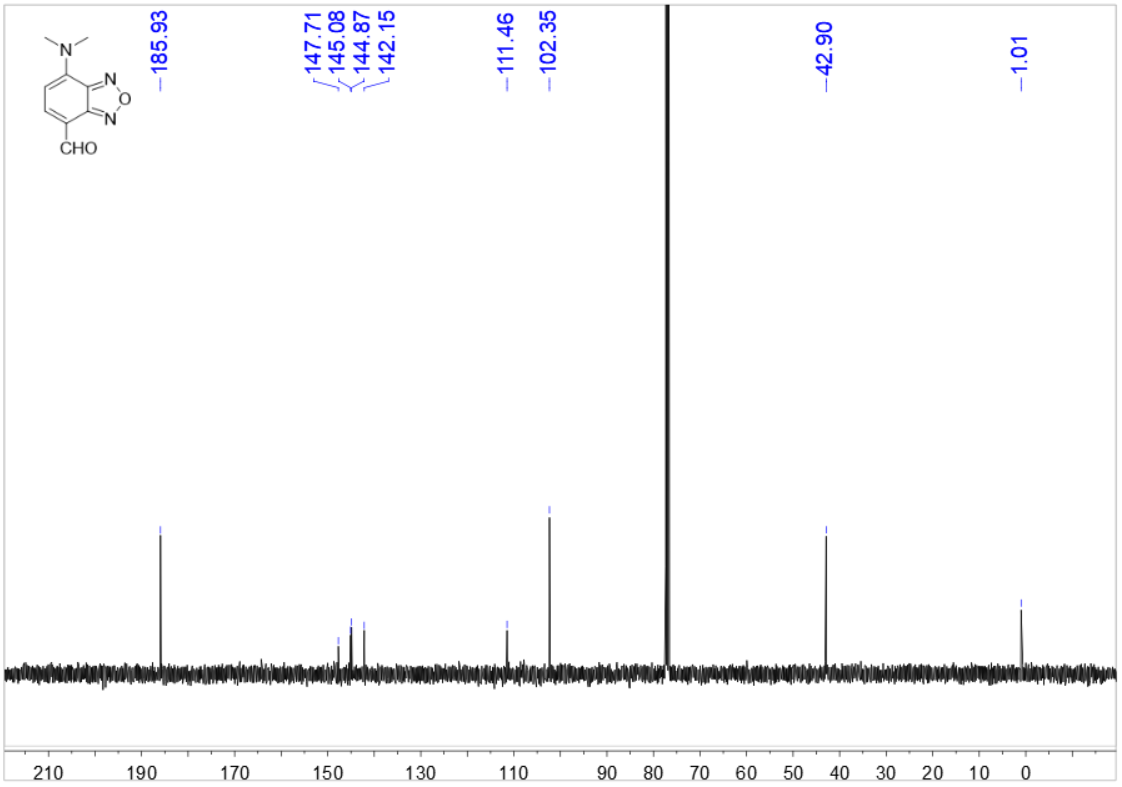


**Figure S14** ^13^C NMR of the compound **2** in CDCl_3_


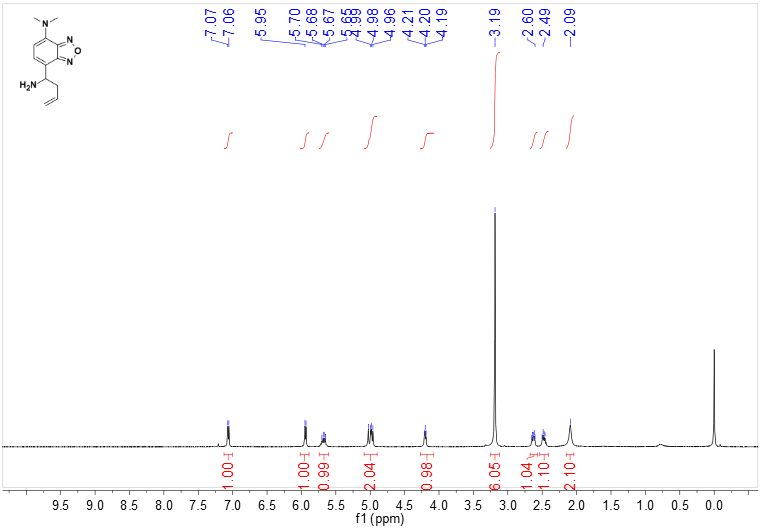


**Figure S 15** ^1^H NMR of the **BD-CHO** in CDCl_3_


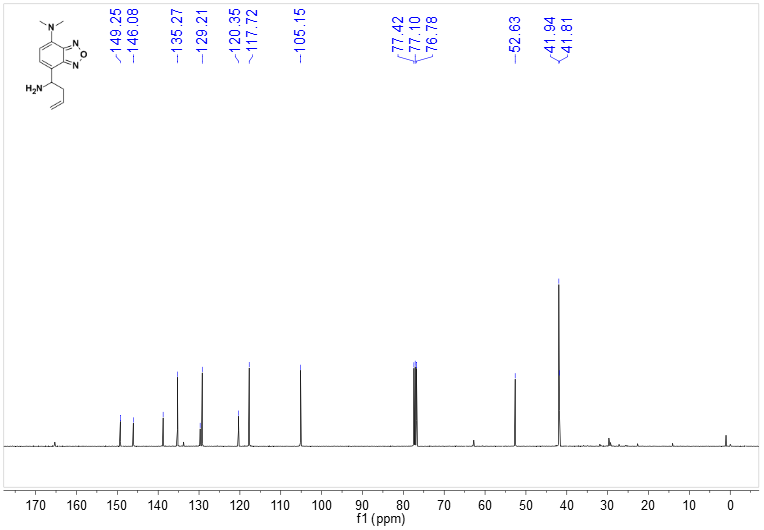


**Figure S 16** ^13^C NMR of the **BD-CHO** in CDCl_3_
